# Relative Contribution of Th1 and Th17 Cells in Adaptive Immunity to *Bordetella pertussis*: Towards the Rational Design of an Improved Acellular Pertussis Vaccine

**DOI:** 10.1371/journal.ppat.1003264

**Published:** 2013-04-04

**Authors:** Pádraig J. Ross, Caroline E. Sutton, Sarah Higgins, Aideen C. Allen, Kevin Walsh, Alicja Misiak, Ed C. Lavelle, Rachel M. McLoughlin, Kingston H. G. Mills

**Affiliations:** 1 Immune Regulation Research Group, School of Biochemistry and Immunology, Trinity Biomedical Sciences Institute, Trinity College Dublin, Dublin, Ireland; 2 Adjuvant Research Group, School of Biochemistry and Immunology, Trinity Biomedical Sciences Institute, Trinity College Dublin, Dublin, Ireland; 3 Host Pathogen Interactions Group, School of Biochemistry and Immunology, Trinity Biomedical Sciences Institute, Trinity College Dublin, Dublin, Ireland; The Pennsylvania State University, United States of America

## Abstract

Whooping cough caused by *Bordetella pertussis* is a re-emerging infectious disease despite the introduction of safer acellular pertussis vaccines (Pa). One explanation for this is that Pa are less protective than the more reactogenic whole cell pertussis vaccines (Pw) that they replaced. Although Pa induce potent antibody responses, and protection has been found to be associated with high concentrations of circulating IgG against vaccine antigens, it has not been firmly established that host protection induced with this vaccine is mediated solely by humoral immunity. The aim of this study was to examine the relative contribution of Th1 and Th17 cells in host immunity to infection with *B. pertussis* and in immunity induced by immunization with Pw and Pa and to use this information to help rationally design a more effective Pa. Our findings demonstrate that Th1 and Th17 both function in protective immunity induced by infection with *B. pertussis* or immunization with Pw. In contrast, a current licensed Pa, administered with alum as the adjuvant, induced Th2 and Th17 cells, but weak Th1 responses. We found that IL-1 signalling played a central role in protective immunity induced with alum-adsorbed Pa and this was associated with the induction of Th17 cells. Pa generated strong antibody and Th2 responses, but was fully protective in IL-4-defective mice, suggesting that Th2 cells were dispensable. In contrast, Pa failed to confer protective immunity in IL-17A-defective mice. Bacterial clearance mediated by Pa-induced Th17 cells was associated with cell recruitment to the lungs after challenge. Finally, protective immunity induced by an experimental Pa could be enhanced by substituting alum with a TLR agonist that induces Th1 cells. Our findings demonstrate that alum promotes protective immunity through IL-1β-induced IL-17A production, but also reveal that optimum protection against *B. pertussis* requires induction of Th1, but not Th2 cells.

## Author Summary

The bacterium *Bordetella pertussis* causes whooping cough, a severe and often lethal respiratory infection in humans. The disease was largely controlled through vaccination with whole cell pertussis vaccines (Pw). However, Pw had side effects and were replaced in developed countries in the 1990s with safer acellular pertussis vaccines (Pa). Unfortunately this has now been linked with a recent resurgence of whooping cough. We have used a mouse model to examine the mechanism of host immunity against *B. pertussis*. We examined the type of immune responses induced with Pa compared with Pw in an attempt to identify its shortcomings and to design a more effective vaccine. Traditionally it had been considered that antibodies mediate protection induced with pertussis vaccines. However, we found that blood lymphocytes, in particular a subpopulation of T cells called Th17 cells that secrete a cytokine called IL-17, play a critical role in host immunity induced by Pa. In contrast, Pw induce Th17 cells but also another T cell subtype called Th1 cells, which are also required for optimum immunity. Finally, we rationally designed a new vaccine using a formulation that induces Th1 cells and found that this was highly effective in conferring protective immunity.

## Introduction

*Bordetella pertussis* is a Gram-negative bacterium that causes whooping cough (pertussis), a severe respiratory tract infection that kills almost 200,000 children annually worldwide. Whole cell vaccines (Pw) introduced in the 1950s significantly reduced the incidence of pertussis but were associated with side effects and were replaced by safer acellular pertussis vaccines (Pa) in most developed countries following successful clinical trials in the 1990s [1]–[3]. However the incidence of pertussis is increasing, especially in adolescents and adults [4], [5] and this may be related to suboptimal or waning immunity induced by Pa [6].

Despite recent progress, the mechanism of protective immunity induced by pertussis vaccines remains unclear. Analysis of serological responses in immunized children revealed a correlation between antibody response to the *B. pertussis* antigens, pertactin, pertussis toxin (PT) or fimbrae and Pa-induced protection [7]. Analysis of T cell responses in children demonstrated that Pa promote Th2-type responses, whereas Pw preferentially induce Th1 cells [8], [9]. Studies in mouse models have suggested that Th1 cells play a critical role in immunity induced by Pw or previous infection, whereas Th2 cells and antibody confer protection induced by Pa [10]–[13]. However it has also been reported that the superior long term protection induced by Pw in mice, when antibody responses had waned significantly, was associated with the induction of potent Th1 responses [14]. More recently it has been reported that Th17 cells also play a role in protection induced by natural infection or immunization with Pw [15]–[18], but their role in Pa-induced immunity has not been examined.

Like most other licensed infectious disease vaccines, Pa are delivered to children using alum as the adjuvant. Traditionally it had been accepted that alum enhances immune responses to the antigens in a vaccine by facilitating retention of the antigen at the site of injection, thus promoting antibody responses and antigen uptake by antigen presenting cells for priming of T cell responses in the draining lymph nodes [19]. It also emerged that alum preferentially promoted Th2 cells, which are considered to be important for protection against parasites and extracellular bacteria by providing help for antibody production. More recently, it was demonstrated that alum functions as an adjuvant in mice by activating the Nlrp3 inflammasome [20], [21], involved in processing of IL-1β. It has also been reported that activation of caspase-1 and Nlrp3, although required for IL-1β production, were dispensable for alum-mediated Th2-associated antibody production [22]. However, the role of Th17 cells has not been addressed.

We and others have shown that caspase-1-processed IL-1β plays a crucial role in the induction of Th17 cells that mediate autoimmunity [23]–[25]. Th17 cells are also required for protective immunity against infection, primarily fungi and extracellular bacteria, such as *Klebsiella pneumonia*, where IL-17 promotes recruitment of neutrophils [26].

The aim of this study was to examine the relative contribution of Th1 and Th17 cells in host immunity to *B. pertussis*, both in the clearance of a primary infection in naive mice and in response to vaccination and to use this information to help in the rational design of a more effective Pa. Our findings demonstrate both Th1 and Th17 cells contribute to clearance of a primary infection of mice with *B. pertussis*, and that IFN-γ has a critical role in adaptive immunity to *B. pertussis* induced by Pw. In contrast, an alum-adjuvanted Pa induced Th17 as well as Th2-type responses, but surprisingly we found that IL-17A played an essential role, while IL-4 was unnecessary for bacterial clearance. The induction of Th17 responses by Pa required activation of IL-1R-signalling in innate immune cells and protection was associated with cellular recruitment to the lungs after challenge with *B. pertussis* and activation of bacterial killing by neutrophils. Furthermore, the protective efficacy of experimental Pa could be enhanced to that of Pw by substituting alum with an adjuvant that induces Th1 cells.

## Results

### Th17 and Th1 cells mediate natural immunity to *B. pertussis*

**Figure 1:**
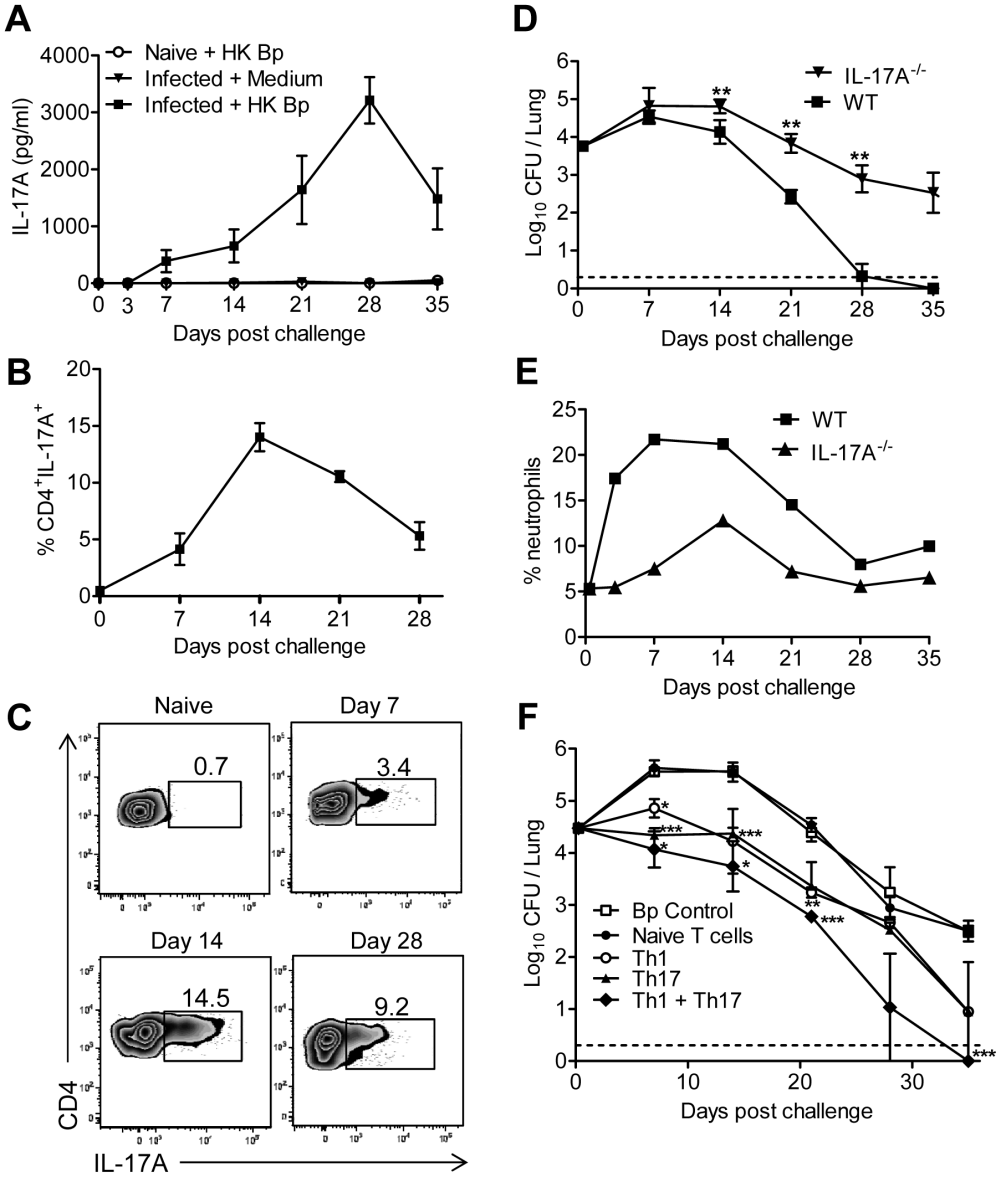
Th17 and Th1 cells mediate host immunity to *B. pertussis* in the respiratory tract of naive mice. (A–C) Naive C57BL/6 mice were exposed to an aerosol infection with *B. pertussis* and groups of 4 mice were sacrificed at the indicated time points. (A) Lung mononuclear cells were stimulated with heat-killed *B. pertussis* and after 3 days of culture IL-17A was quantified in supernatants by ELISA. (B–C) lung mononuclear cells were incubated with brefeldin-A for 1 h and intracellular cytokine staining for IL-17A, together with surface staining for CD4 was performed, followed by FACS analysis. Results are expressed as mean frequencies of IL-17A^+^CD4^+^ cells (B), with sample FACS plots (C) (D–E) C57BL/6 WT and IL-17A^−/−^ mice were aerosol challenged with *B. pertussis* and groups of 4 mice were sacrificed at the indicated time points. CFU counts were performed on lung homogenates (D) ** p<0.01 IL-17A^−/−^ versus WT. Neutrophil recruitment was determined by FACS analysis on lung lavage (E). (F) Spleen cells from IFN-γ^−/−^ or WT mice that had cleared a respiratory infection with *B. pertussis* were stimulated *in vitro* with killed *B. pertussis* and IL-12 (Th1) or IL-1β and IL-23 (Th17) respectively. After 4 days of culture surviving cells were harvested and *B. pertussis*-specific Th1, Th17 or both (10×10^6^) were transferred to naive mice, which were aerosol challenged with live *B. pertussis* 24 hours later. Naive mice that did not receive a cell transfer and mice injected with T cells from a naive mouse were used as controls. The course of infection was followed by performing CFU counts on the lungs at intervals after challenge. +p<0.05, +++ p<0.001 Th1+Th17 versus control; ** p<0.01, *** p<0.001 Th17 versus control. Results (except panel C) are mean values for 4 mice per group at each time point and each panel is representative of either 3 to 4 independent experiments.

Previous infection with *B. pertussis* is effective in inducing protective immunity against subsequent infection and this has been associated with the induction of Th1 cells [13], [27]. Indeed, it has already been established that IFN-γ plays a critical role in clearance of a primary infection with *B. pertussis* [10], [11]. However there is also evidence that Th17 cells may be involved [16], [18]. Here we examined the relative role of T cell subtypes in host immunity to a primary infection with *B. pertussis* in naive mice and first concentrated on defining the role of IL-17. We found that infection of mice with *B. pertussis* was associated with induction of *B. pertussis*-specific Th17 cells. Antigen-specific IL-17A (Figure 1A) and IL-17F (Figure S1A) production was detected in lungs as early as 7 days post challenge and reached a peak after 3–4 weeks. Interestingly, *B. pertussis* filamentous hemagglutinin (FHA), which is considered to be the least important antigen in Pa from the perspective of antibody responses [7], was a major target for Th17 cells from infected mice (Figure S1B). In order to confirm these findings and to examine the cellular source of IL-17, we performed intracellular cytokine staining (ICS) and flow cytometry analysis on lung mononuclear cells *ex vivo*, without re-stimulation. We found significant increase in the frequency (Figure 1B, C) and absolute numbers (Figure S2) of IL-17A-producing CD4 T cells in the lung throughout the course of infection with *B. pertussis*. The earlier peak of IL-17A^+^CD4^+^ T cells (day 14) compared with antigen-specific IL-17A detected by ELISA (day 21), probably reflect the difference in the assay system, with the latter involving a re-stimulation *in vitro* and therefore including memory cells, while the ICS was a more direct *ex vivo* measure of activated effector Th17 cells. Taken together these data show that *B. pertussis* infection of mice induces significant numbers of *B. pertussis*-specific Th17 cells in the lungs.

In order to examine the role of IL-17A in bacterial clearance, we compared the course of infection in IL-17A-defective (IL-17A^−/−^) and WT mice. IL-17A^−/−^ mice had 100–1000 fold more CFU in the lungs at the later stages of infection with bacteria still detectable in the lungs up to week 6 (Figure 1D). The more severe infection in IL-17A^−/−^ mice was associated with a significant reduction in CXCL1 (KC) production (Figure S3) and impaired neutrophil recruitment (Figure 1E) to the lungs post challenge.

We used an adoptive cell transfer approach to examine the relative role of Th1 and Th17 cells in protective immunity to *B. pertussis*. We generated polarized *B. pertussis*-specific Th1 or Th17 cells (Figure S4) by culture of spleen cells from convalescent WT or IFN-γ^−/−^ (to overcome the problems of reversion of Th17 cells to Th1) with antigen and IL-12 or IL-1β and IL-23 respectively. Transfer of either Th1 or Th17 cells alone before *B. pertussis* challenge reduced the CFU counts by about 10 fold over the course of infection (Figure 1F). Transfer of both populations together had a greater effect with CFU count significantly reduced by 50–100 fold compared to controls. In contrast transfer of naïve T cells from WT mice failed to confer protection to infected mice. These findings demonstrate that both Th1 and Th17 cells contribute to natural immunity induced by infection with *B. pertussis* in mice.

### Protective immunity induced by Pw is mediated largely by IFN-γ production

**Figure 2:**
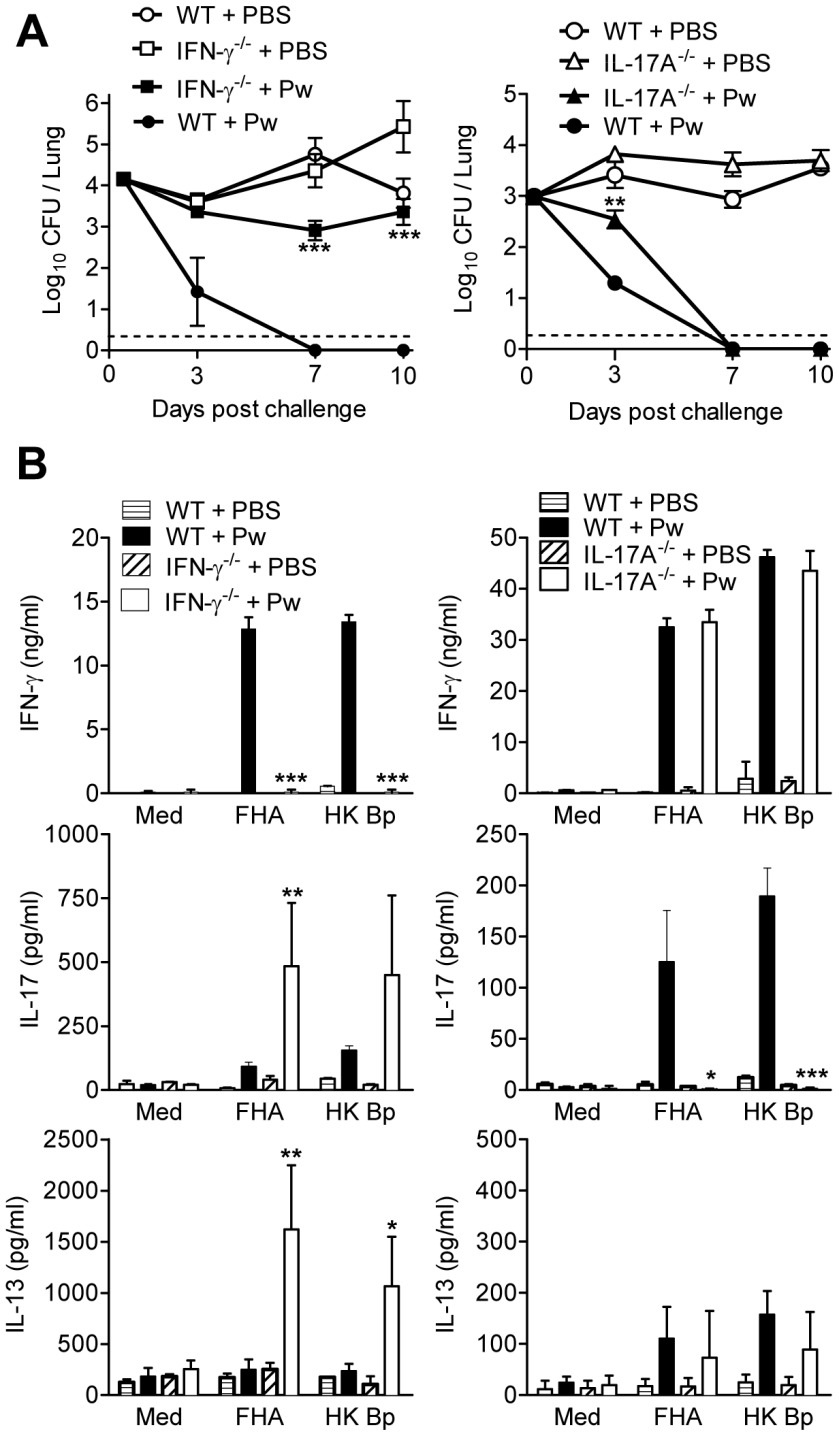
Protective immunity induced with Pw is mediated by IFN-γ and IL-17. WT, IFN-γ^−/−^ or IL-17A^−/−^ mice were immunized i.p. twice (0 and 28 days) with Pw. 14 days after the second immunization, mice were challenged by exposure to an aerosol of live *B. pertussis*. (A) The number of CFU in the lungs were quantified at intervals after challenge. (B) *B. pertussis*-specific cytokine production by spleen cells on day of challenge. *p<0.05, **p<0.01, ***p<0.001 IFN-γ^−/−^ or IL-17A^−/−^ versus WT. Results are mean values for 4 mice per group at each time point and each panel is representative of 2 independent experiments.

Pw are more protective than Pa in mice [12], [13], [28], [29], which is even more pronounced when mice are challenged at an extended interval after immunization [14] and this has been attributed to the induction of Th1 cells by Pw [13]. Although a Connaught laboratories Pw only had an efficacy of 36 or 48% compared with 84 or 85% for 3 and 5-component Pa in the pertussis clinical trials carried out in Sweden and Italy in the 1990s [1], [2], most good Pw have efficacy of 93–96% in children [3], [30], [31] and a UK Pw was significantly more protective than the three-component Pa in a randomized controlled trial [32]. Here we examined the relative roles of IFN-γ and IL-17 in clearance of *B. pertussis* from the respiratory tract of mice immunized with Pw. We used a plain (without alum) Pw reference preparation. Although most recent Pw are absorbed to alum, plain Pw, such as the one manufactured by Wellcome laboratories, were routinely used until the 1980s in many European countries and had high efficacy against pertussis [30], [33]. Furthermore, we have found that plain Pw induce similar immune responses and protection against infection as alum-absorbed Pw [12] [and Mills, unpublished]. Here we found that protective immunity induced by Pw was significantly compromised in IFN-γ^−/−^ mice, with 100–1000 fold more bacteria in the lungs compared with Pw-immunized WT mice 3, 7 and 10 days after aerosol challenge (Figure 2A). The CFU counts were also significantly higher in Pw-immunized IL-17A^−/−^ compared with WT mice 3 days post *B. pertussis* aerosol challenge, but IL-17A^−/−^ mice, like WT mice had cleared the infection by day 7.

Mice immunized with Pw developed strong Th1 responses with high concentrations of IFN-γ produced by spleen cells from Pw immunized WT and IL-17A^−/−^ mice, which was undetectable in IFN-γ^−/−^ mice (Figure 2B). *B. pertussis*-specific IL-17 was also induced by Pw and this was enhanced in IFN-γ^−/−^ mice. *B. pertussis*-specific IL-13 was at background concentrations in spleen cells from Pw-immunized WT mice, but was induced at significant concentrations in IFN-γ^−/−^ mice (Figure 2B). These findings demonstrate that Pw induce Th1 and Th17 cells and confer protective immunity in mice via IFN-γ induction, but that IL-17A also contributes, though less significantly.

### Protective immunity induced by Pa is dependent on IL-17A but not IL-4 or IFN-γ

**Figure 3:**
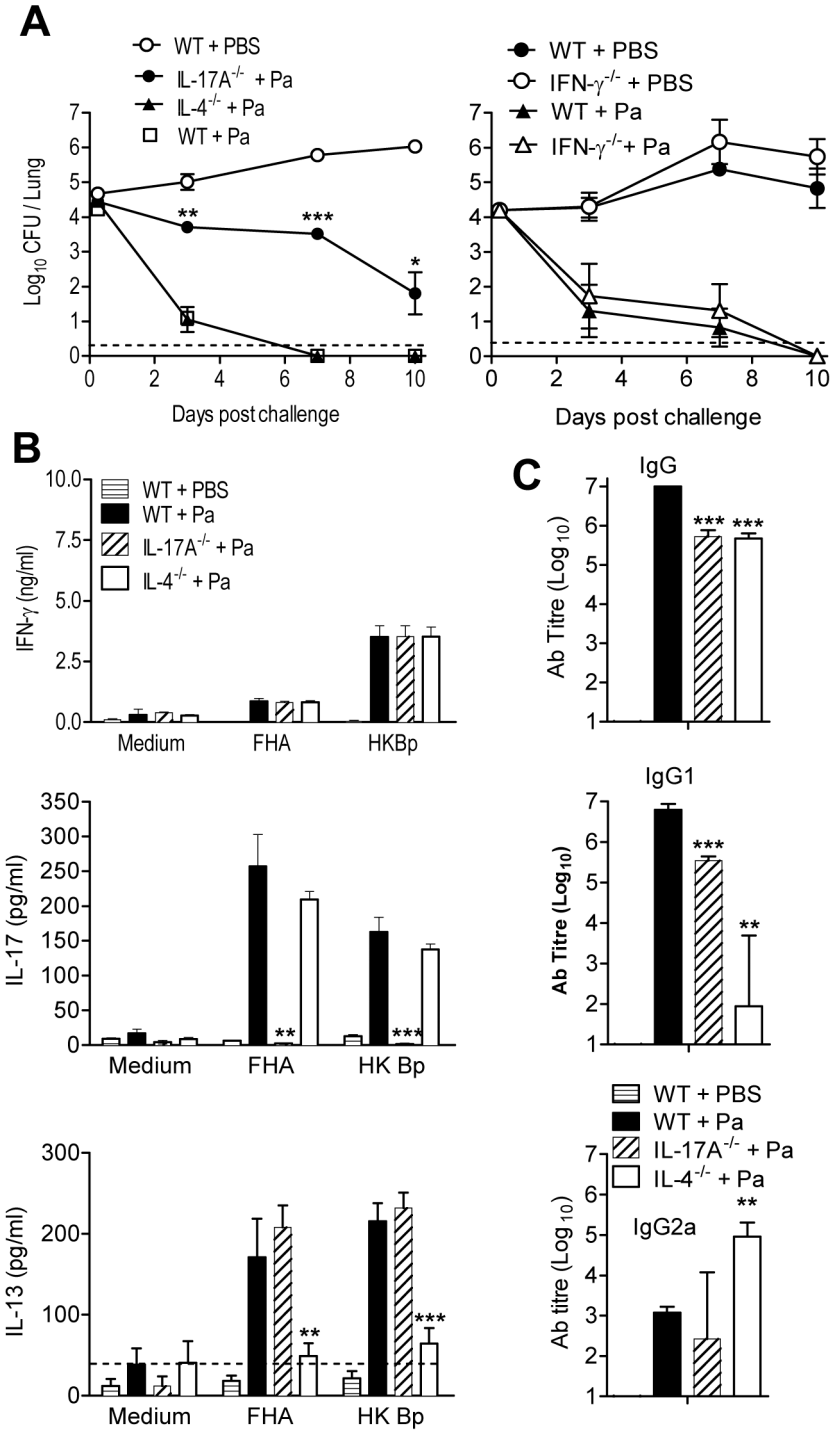
Protective immunity induced with Pa is dependent on IL-17A but not IL-4. WT, IL-17A^−/−^, IL-4^−/−^ or IFN-γ^−/−^ mice were immunized i.p. twice (0 and 28 days) with Pa. 14 days after the second immunization, mice were challenged by exposure to an aerosol of live *B. pertussis*. (A) The number of CFU in the lungs were quantified at intervals after challenge. (B) *B. pertussis*-specific cytokine production by spleen cells on day of challenge. (C) *B. pertussis*-specific antibody in serum on the day of challenge. *p<0.05, **p<0.01, ***p<0.001 knockout versus WT. Results are mean values for 4 mice per group at each time point and each panel is representative of 2 independent experiments.

Having shown that protection induced by Pw is mediated largely by Th1 cells, we examined the mechanism of host immunity induced by immunization with a licensed alum-absorbed Pa. Immunization with Pa by either i.p. or i.m. routes conferred protection against *B. pertussis* infection (Figure S5A). We have previously reported that Pa selectively induced Th2-type responses whereas Pw promoted Th1 responses [12], [13]. Here we found that Pa also induced *B. pertussis*-specific IL-17A from CD4^+^ T cells (Figure S5B). We next examined the role of Th17 versus Th1 and Th2 cells in Pa-induced immunity. The bacterial clearance curves were almost identical for Pa-immunized WT and IL-4^−/−^ or IFN-γ^−/−^ mice (Figure 3A). In contrast, the rate of bacterial clearance was dramatically slower in IL-17A^−/−^ mice, with 100 fold more bacteria on day 3 and significant bacteria in the lungs on day 10, when the WT mice had cleared the infection (Figure 3A). Pa still induced Th2 responses in IL-17A^−/−^ mice, with *B. pertussis*-specific IL-13 similar to that in WT mice (Figure 3B). In contrast, *B. pertussis*-specific IL-13 production by spleen cells was close to background concentrations in IL-4^−/−^ mice, whereas IL-17 was similar to that seen in Pa-immunized WT mice. FHA-specific IFN-γ was undetectable in Pa immunized mice and the low levels of IFN-γ detected in response to HKBp was not significantly different between WT, IL-17A^−/−^ and IL-4^−/−^ mice (Figure 3B). Collectively these findings demonstrate an essential role for IL-17A, but not for IL-4 or IFN-γ, in protective immunity induced by Pa in mice.

An examination of antibody responses revealed that total IgG and IgG1 were significantly reduced in both IL-4^−/−^ and IL-17A^−/−^ mice (Figure 3C). IgG2a (Figure 3C) and IgG2c (data not shown) were significantly higher in IL-4^−/−^ than WT mice, but similar in IL-17A^−/−^ and WT mice.

**Figure 4:**
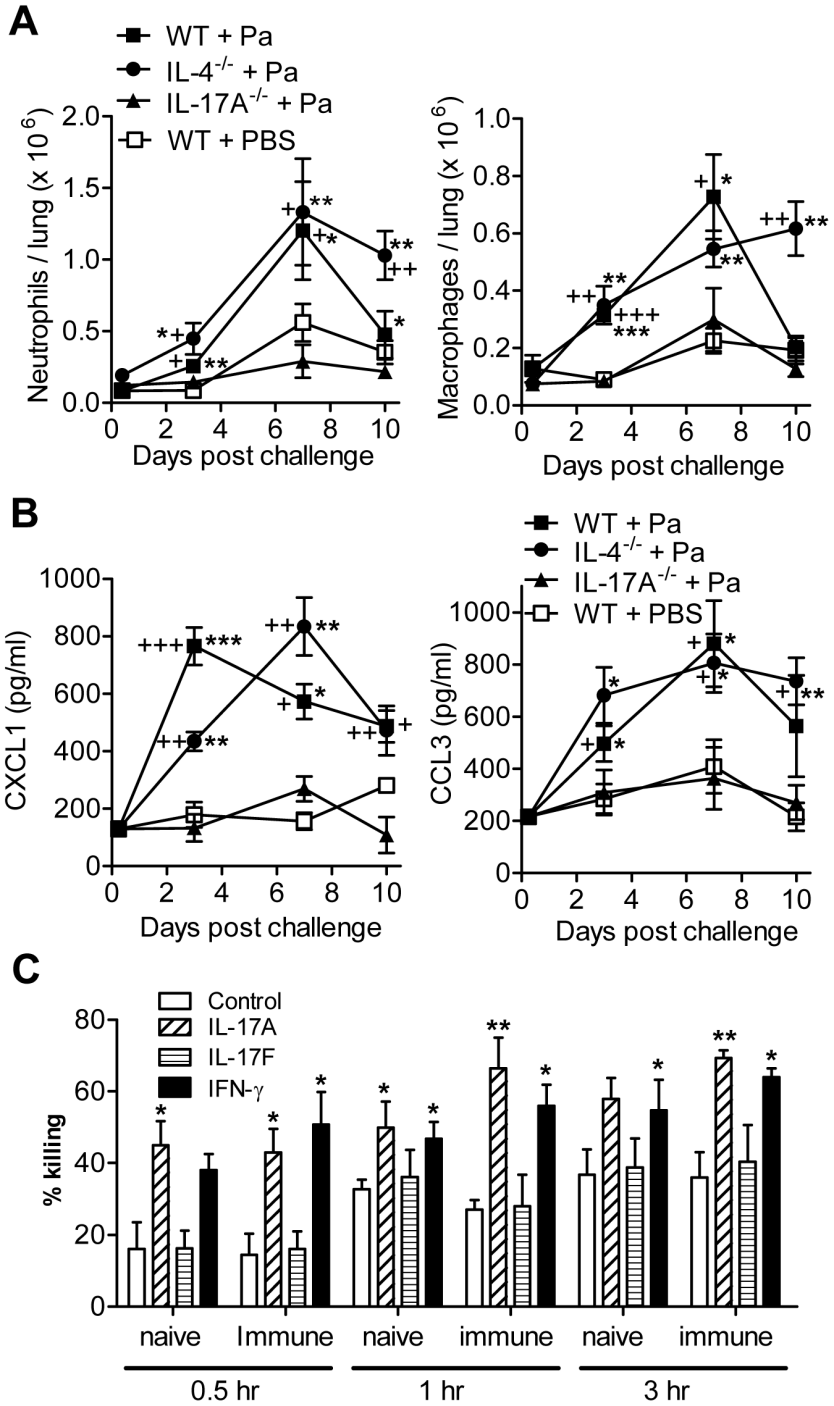
Induction of protective Th17 cells is associated with neutrophil recruitment and killing of *B. pertussis*. (A, B) WT, IL-4^−/−^ and IL-17A^−/−^ mice were immunized i.p. with Pa and challenged with *B. pertussis* as described in Figure 3. Recruitment of GR1^+^ neutrophils and F4/80^+^ macrophages in the lungs (A) and CXCL1 and CCL3 concentrations in lung homogenates (B) following aerosol challenge with live *B. pertussis*. p<0.05, **p<0.01, ***p<0.001 WT + Pa or IL-4^−/−^ + Pa versus WT + PBS; +p<0.05, ++ p<0.01, +++ p<0.001 WT + Pa or IL-4^−/−^ +Pa versus IL-17A^−/−^ +Pa. (C) Effect of recombinant IL-17A, IL-17F or IFN-γ, in the presence of mouse serum from naive or immune mice (containing *B. pertussis* antibodies from Pa-immunized mice) on neutrophil-mediated killing of *B. pertussis in vitro*. *p<0.05, **p<0.01 versus control. Results in A and B are mean values for 4 mice per group at each time point and each panel is representative of 3 independent experiments. Results in C are mean values for triplicate assays and are representative of 3 experiments.

To examine the mechanism of immune protection mediated by IL-17A in the lungs, we investigated phagocytic cell influx upon *B. pertussis* challenge. There was a significant increase in the recruitment of both neutrophils and macrophages to the lungs after *B. pertussis* challenge in Pa-immunized mice compared to non-immunized mice, which peaked at day 7 post challenge (Figure 4A). Cellular recruitment to the lungs was similar in Pa-immunized WT and IL-4^−/−^ mice. In contrast, the influx of neutrophils and macrophages was significantly reduced in Pa-immunized IL-17A^−/−^ mice. This was associated with dramatically lower CXCL1 and CCL3 (MIP-1α) concentrations in the lungs of Pa-immunized IL-17A^−/−^ compared with WT or IL-4^−/−^ mice post challenge (Figure 4B).

We have previously reported that IL-17 can promote macrophage killing of *B. pertussis* [15]. Here we demonstrate that neutrophils were also capable of killing *B. pertussis* following opsonisation with normal mouse serum, with killing detected after 1–3 hours, and this was significantly enhanced by IL-17A or IFN-γ but not IL-17F (Figure 4C). Furthermore, killing was further, though not significantly, enhanced following addition of immune serum from Pa-immunized mice (Figure 4C). These findings suggest that Pa-induced IL-17A enhances chemokine production, which recruits macrophages and neutrophils to the lungs soon after challenge with *B. pertussis* and these cells mediate killing of *B. pertussis*. Contrary to the perceived wisdom, our study suggests that Th2 cells are unnecessary, and Th17 cells play a critical role in protective immunity induced with Pa.

### Pa promotes the induction of Th17 cells via IL-1

We examined the mechanism of Th17 cell induction with Pa, in particular the role of Nlrp3 and IL-1. It had previously been reported that alum functions as an adjuvant by activation of the Nlrp3 inflammasome [20], [21], although this has been questioned by others [22]. Furthermore, we had previously shown that IL-1β, induced via caspase-1 and Nlrp3, plays a critical role in IL-17-mediated pathology in autoimmune disease [23], [25], [34]. Here we found that Pa or alum induced significant concentrations of IL-1β from LPS-primed DC and this was significantly reduced following addition of a caspase-1 inhibitor (Figure S6A) or using DC from Nlrp3^−/−^ mice (Figure S6B). Furthermore, significant concentrations of IL-1β were detected in draining lymph nodes 4 hours after injection of Pa (Figure S6C). These finding demonstrate that alum-absorbed Pa promotes IL-1β production by DC *in vitro* via activation of caspase-1 and Nlrp3.

We also examined the role of Nlrp3 in the protective efficacy of Pa *in vivo*. Bacterial clearance was reduced though only significantly on day 5 post challenge in Pa-immunized Nlrp3^−/−^ compared with WT mice (Figure S7A). Furthermore, IL-17A production determined by ELISA on *B. pertussis* antigen-stimulated spleen cells (Figure S7B) or by intracellular cytokine staining on CD4^+^ T cells (Figure S7C) was similar in Pa-immunized Nlrp3^−/−^ and WT mice. Finally IL-1β production in the lungs of *B. pertussis* infected mice was not significantly different between Nlrp3^−/−^ and WT mice (Figure S7D).

**Figure 5:**
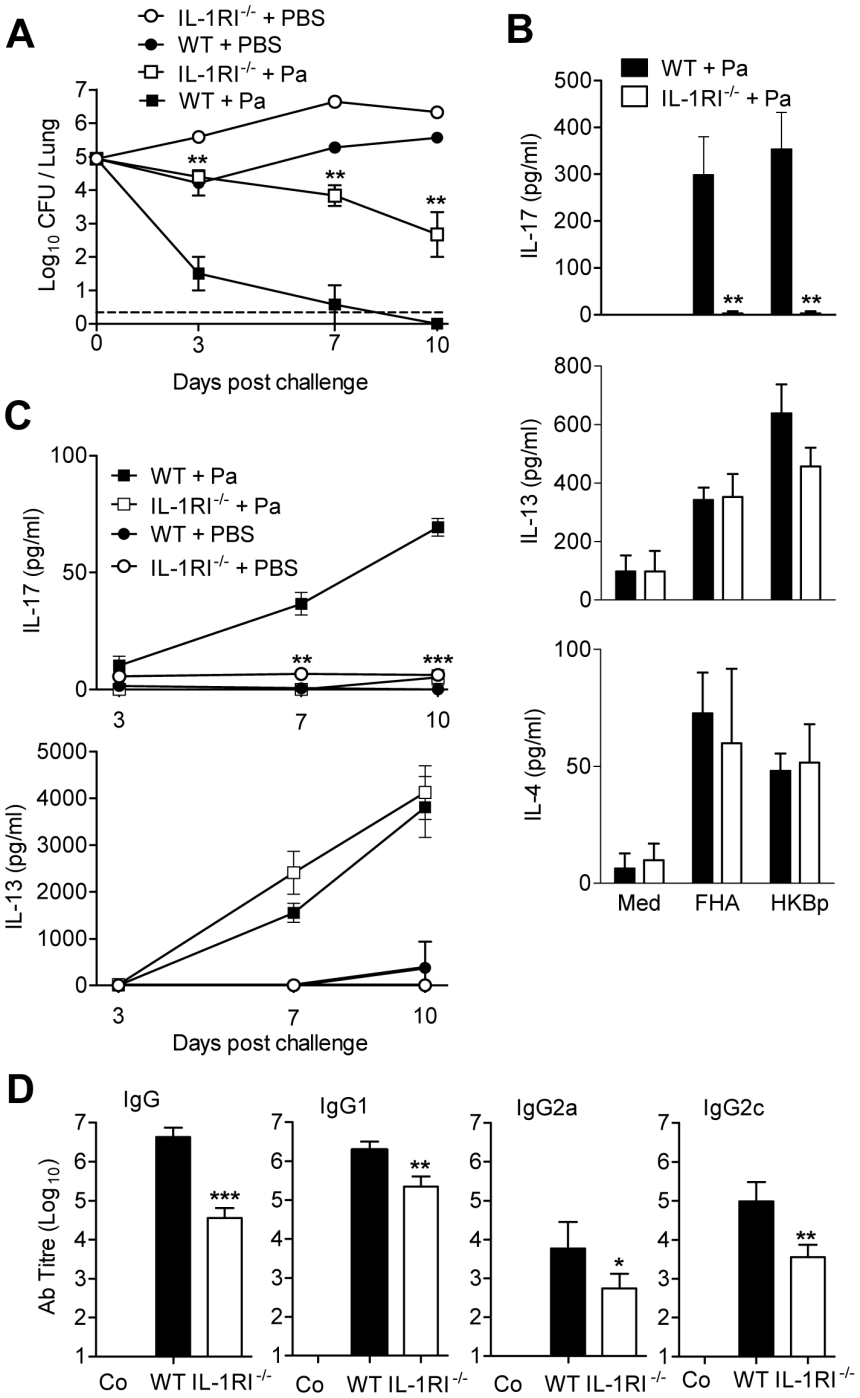
IL-1RI signalling is required for induction of Th17 responses and Pa-induced protection against *B. pertussis*. IL-1RI^−/−^ and WT mice were immunized i.p. twice (0 and 28 days) with Pa. 14 days after the second immunization, mice were challenged by exposure to an aerosol of live *B. pertussis*. (A) The number of CFU in the lungs were quantified at intervals after challenge. (B, C) *B. pertussis*-specific cytokine production by spleen cells on the day of challenge (B) or *B. pertussis*-specific cytokine production by lung mononuclear cells 3, 7 and 10 days post challenge (C) was determined by ELISA. (D) *B. pertussis*-specific antibody in serum on the day of challenge (Co: control; KO: IL-1RI^−/−^). *<p0.05, **p<0.01, ***p<0.001 IL-1RI^−/−^ versus WT. Results are mean values for 4 mice per group at each time point and each panel is representative of 3 independent experiments.

In contrast to the rather limited attenuation of anti-*B. pertussis* immunity in Pa-immunized Nlrp3^−/−^ mice, we found a dramatic reduction in the rate of bacterial clearance in Pa-immunized IL-1RI^−/−^ mice, with 1000 fold more bacteria in the lungs when compared with Pa-immunized WT mice at 3 and 7 days post challenge (Figure 5A). Furthermore, WT mice had completely cleared the bacteria by day 10, where as there were significant numbers of bacteria in the lungs of IL-1RI^−/−^ mice at this time point. These findings demonstrate that IL-1 is critical for protection, and its induction *in vitro* is dependent on caspase-1 and NLRP3, but *in vivo* NLRP3 appears to be dispensable, suggesting that NLPR3-independent IL-1 pathways may be involved.

Immunization of WT mice with Pa induced strong Th2-type responses and Th17 responses. However *B. pertussis*-specific IL-17A production was undetectable in spleen cells from IL-1RI^−/−^ mice immunized with Pa (Figure 5B). In contrast, IL-4 and IL-13 production was similar in Pa-immunized WT and IL-1RI^−/−^ mice. In addition, *B. pertussis*-specific IL-17 was detectable in lungs 7 and 10 days after challenge of WT mice immunized with Pa, but was completely undetectable in IL-1RI^−/−^ mice (Figure 5C). In contrast, significant concentrations of IL-13 were detected in the lungs 7 and 10 days after *B. pertussis* challenge of WT and IL-1RI^−/−^ mice immunized with Pa (Figure 5C).

Immunization of mice with Pa induced potent antibody responses, predominantly of the IgG1 subclass; this was significantly reduced in IL-1RI*^−/−^* mice (Figure 5D). Pa generated weaker IgG2a and IgG2c antibody responses, which were also reduced in IL-1RI^−/−^ mice. These findings demonstrate that IL-1 signalling plays an essential role in Pa-induced immunity and this involves induction of Th17 cells and antibody, but not Th2 cells.

### An adjuvant that promotes Th1 and Th17 cells enhances efficacy of Pa

**Figure 6:**
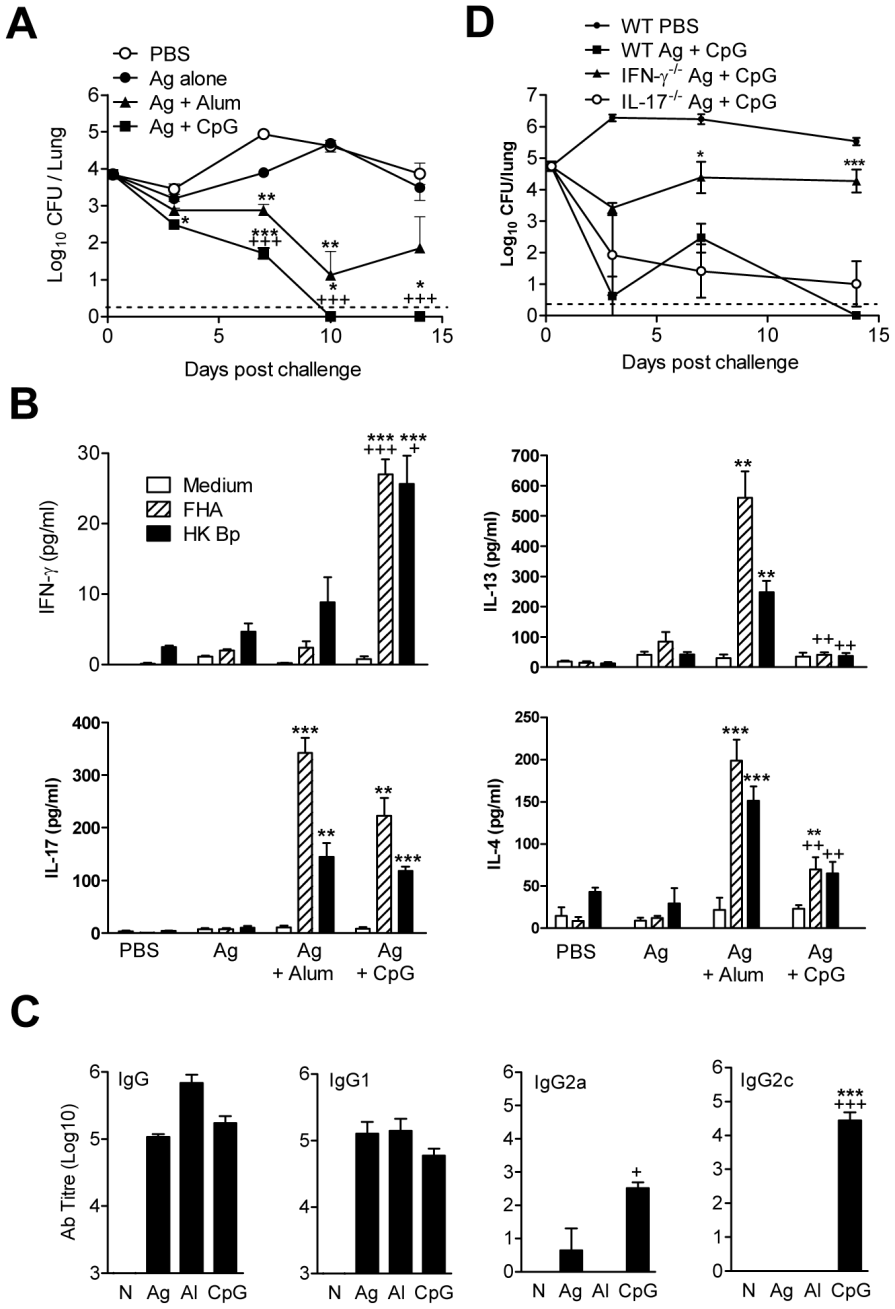
Substitution of CpG for alum promotes induction of Th1 cells, which enhances the efficacy of a laboratory-prepared pertussis vaccine. Mice were immunized i.p. twice (0 and 28 days) with PBS, laboratory-prepared Pa in PBS (Ag) or formulated with alum (Al) or CpG. Mice were challenged by exposure to an aerosol of live *B. pertussis* 14 days after the second immunization. (A) The number of CFU in the lungs were quantified at intervals after challenge. (B) *B. pertussis*-specific cytokine production by spleen cells on day of challenge. (C) *B. pertussis*-specific antibody in serum on the day of challenge. +p<0.05, ++p<0.01, +++p<0.001 CpG versus alum; *p<0.05, **p<0.01, ***p<0.001 versus antigen in PBS. (D) WT, IL-17A^−/−^ or IFN-γ^−/−^ mice were immunized i.p. twice with a laboratory-prepared Pa formulated with CpG. Mice were challenged by exposure to an aerosol of live *B. pertussis* 14 days after the second immunization. The number of CFU in the lungs were quantified at intervals after challenge. Results are mean values for 4 mice per group at each time point and each panel (except D) is representative of 2 independent experiments.

Since previous infection, and immunization with Pw induce potent Th1 cells and confer high levels of protection against *B. pertussis* [12]–[14], [28], [29] (and present study), we examined the hypothesis that the efficacy of Pa could be enhanced by substituting alum with an adjuvant such as CpG, which promotes Th1 cells [35]. Since commercially available vaccines are already adsorbed to alum, here we used a laboratory prepared vaccine composed of the two *B. pertussis* antigens used in all licensed Pa, FHA and detoxified PT. Immunization of mice with the antigens (Ag) alone without adjuvant failed to confer immunity against *B. pertussis* infection (Figure 6A). Consistent with our earlier studies, Ag formulated with alum conferred a good level of protection, however, this was significantly enhanced when the Ag were formulated with CpG. Bacteria were undetectable on days 10 and 14 post challenge in mice immunized with Ag and CpG, but were still detectable in mice immunized with Ag and alum (Figure 6A).

An examination of immune responses on the day of challenge revealed that Ag formulated with CpG induced potent *B. pertussis*-specific IFN-γ production and also induced IL-17A, but low concentrations of IL-4 and IL-13 (Figure 6B). In contrast, immunization with Ag and alum generated *B. pertussis* T cells that secreted IL-4 and IL-13, as well as IL-17A but little IFN-γ. The strongest IgG response was induced with alum as the adjuvant and these antibodies were almost exclusively IgG1 (Figure 6C). Surprisingly, immunization with Ag alone did induce significant IgG1 antibody, but weak T cell responses. In contrast, CpG induced modest IgG1, but significantly higher IgG2a and IgG2c titres than that detected with alum as the adjuvant. These findings demonstrate that switching the adjuvant from alum to CpG promotes the induction of Th1, Th17 and IgG2 rather than Th2, Th17 and IgG1 responses.

In order to examine the relative contribution of IL-17A and IFN-γ in protection induced by Ag administered with CpG, we performed immunization and challenge experiments in WT, IL-17A^−/−^ and IFN-γ^−/−^ mice. The results revealed that WT and IL-17A^−/−^ mice immunized with Ag and CpG effectively cleared the bacteria after *B. pertussis* respiratory challenge (Figure 6D). In contrast, clearance was significantly compromised in IFN-γ^−/−^ mice, with 100–1000 fold more bacteria in immunized IFN-γ^−/−^ when compared with WT or IL-17A^−/−^ mice (Figure 6D), demonstrating the key role of IFN-γ but not IL-17A in immunity induced with CpG as the adjuvant.

Our findings point to a key role for Th1 and Th17 cells in immunity induced by pertussis vaccines formulated with CpG and alum respectively. We and others have reported that IFN-γ-producing cells play a critical role in host immunity to *B. pertussis* during primary infection in part by activating macrophages to kill intracellular bacteria [10], [11], [36]. Here we found that mice immunized with Ag and CpG had significantly more macrophages in their lungs than non-immunized mice 3 days post *B. pertussis* respiratory challenge (Figure S8). This enhanced macrophage recruitment was lost in Ag and CpG-immunized IFN-γ^−/−^ but not in IL-17A^−/−^ mice (Figure S8), providing indirect evidence that immunization with pertussis antigens in combination with a Th1-promoting adjuvant promote recruitment of macrophages to the lungs post challenge with *B. pertussis*. Collectively our findings demonstrate that Th1 cells play a more critical role than Th17 or Th2 cells in host immunity to *B. pertussis* and have significant implication for the rational design of more effective Pa.

## Discussion

The significant new finding of this study is that Th17 cells mediate protective immunity induced with current alum-adjuvanted Pa. In contrast, immunity induced by infection or immunization with Pw is mediated largely by Th1 cells, with a smaller contribution from Th17 cells. Although Th2 cells are strongly induced by Pa in mice and humans, and are considered to be important in promoting antibody responses to extracellular pathogens, our study demonstrated that they are not necessary for protective immunity in a mouse model.

Using a mouse respiratory challenge model, we demonstrate that transfer of Th1 or Th17 cells prior to infection of naive mice reduced the bacterial burden post challenge. We had previously reported that mice defective in IFN-γR develop disseminating lethal infection following primary challenge with *B. pertussis* [10], and here we show that the bacterial burden and clearance is also significantly compromised in IL-17A^−/−^ mice. Collectively, these studies suggest that both Th1 and Th17 cells function in natural immunity to *B. pertussis*. Immunization with Pw also induced Th1 and Th17 responses but studies in IFN-γ^−/−^ mice demonstrated a dominant role for Th1 cells in mediating vaccine induced protection. In contrast a licensed Pa induced *B. pertussis*-specific Th17 and Th2 cells but failed to generate Th1 cells. Challenge experiments in cytokine defective mice demonstrated that IL-4 was dispensable, whereas IL-1 and IL-17A were absolutely required for protective immunity induced with Pa. Furthermore, we demonstrate that protection induced by immunization of mice with genetically detoxified PT and FHA could be enhanced by substituting alum with an adjuvant that induces Th1 as well as Th17 cells.

All animal models involving inbred mice, including the one used in this study have some limitations in terms of extrapolating of experimental findings to humans. Nevertheless, we have shown that the rate of bacterial clearance in mice immunized with Pa relative to control non-immunized mice, correlates with vaccine efficacy in children [12]. Furthermore, there are significant parallels in the T cell responses in mice and humans induced by immunization or infection with *B. pertussis* and these can be summarized as follows: 1) Infection with *B. pertussis* induces Th1, but not Th2, responses in mice [10], [27] and in humans [37]–[40] and also induces Th17 responses in both species [16], [17], [41] [and current study], 2) Immunization with Pw induces Th1 but not Th2 responses in mice [12], [13], [28] and humans [8], [9], [42] and 3) immunization with Pa promotes or enhances Th2 responses in mice [12], [13], [28] and humans [8], [9], [42], [43]. Pa also promote Th17 responses in mice [current study], but this has not yet been fully evaluated in humans. The only slight discrepancy between mouse and human studies is that the responses induced by vaccination with Pa are mixed Th1/Th2 in human and more Th2-dominated in mice, with some reports that Pa induce Th1 responses in humans [44]. However, it has been suggested that the Th1 responses detected in certain Pa-immunized humans may result from natural acquisition following exposure to live *B. pertussis* [37], [39]. Furthermore, booster immunization of 4–6 year old children primed with Pa as infants enhanced Th2 but not Th1 responses [42], [45], suggesting that Pa do promote similar responses in both mice and humans.

Prior to the present study, the consensus view on the mechanism of protection against *B. pertussis* was that like vaccines against other extracellular bacteria, antibodies and Th2 responses played a central role in protective immunity generated by Pa in mice and in humans. Repeated booster immunization with Pa induces very strong *B. pertussis*-specific Th2 [42], [45] and strong but transient serum IgG antibodies in children [46] and mice [14] and correlative studies showed an association between serum antibody response to PT, pertactin and fimbrae 2/3 and protection in children [7]. However, the present study shows that Th2 responses are dispensable for immunity induced by Pa in mice and that IL-17A plays an essential role in protection induced with alum-adjuvanted Pa. Furthermore, FHA, which is considered to be the least important antigen from the perspective of antibody production [7], was a major target for Th17 cells.

It has previously been reported that Th17 cells are involved in adaptive immunity to *Pseudomonas aeruginosa* [47], *Staphylococcus aureus, Candida albicans* [48] and *Helicobacter pylori* [49]. It has also been reported that Th17 cells mediate heterologous protection against *Klebsiella pneumonia* induced by nasal immunization with heat-killed bacteria [50]. In addition, it has been demonstrated that immunization of mice with the *Mycobacterium tuberculosis* ESAT-6 peptide mixed with the TLR4 agonist MPL in combination with trehalose dimycolate analog as the adjuvant/delivery system induced IL-17-producing cells which conferred protection by helping to recruit IFN-γ-secreting Th1 cells to the lungs [51]. However, to our knowledge this is the first study to identify a role for IL-17A in protective immunity against *B. pertussis* in mice induced with a licensed alum-adjuvanted human vaccine.

The induction and expansion of Th17 cells involves a number of inflammatory cytokines, including IL-1β and here we found that alum-adjuvanted Pa or alum alone promoted innate IL-1β production. It has been reported that IL-1β, which is processed by caspase-1, did not mediate the adjuvant activity of alum *in vivo*, however these conclusions were based on the premise that alum promotes immunity via the induction of Th2 and antibody responses. It has been demonstrated that mice defective in caspase-1 or Nlrp3 had reduced Th2 and antibody responses to antigens administered with alum [20], [21]. However, it was later reported that while the induction of IL-1β by alum was dependent on Nlrp3, the enhancement of antibody responses with alum *in vivo* were independent of Nlrp3 [22], [52]. The present study demonstrated that Nlrp3 was required for the induction of IL-1β production by DC *in vitro* with alum or alum-containing Pa, but only played a minor role in the induction of IL-1β, IL-17 and protective immunity induced with Pa *in vivo*, suggesting involvement of Nlrp3-independent pathways in cells other than DCs. This is consistent with a role for Nlrp3 and caspase-1 independent IL-1β in host defences against *Mycobacterium tuberculosis in vivo*. [53], [54]. Consistent with a previous report [55], we found Pa-immunized IL-1RI^−/−^ mice had a significantly higher bacterial burden after *B. pertussis* challenge than WT mice and this was associated with significantly reduced antigen-specific IL-17A production. Collectively these findings suggest that the adjuvant activity of alum in enhancing the immunogenicity of Pa is mediated at least in part by induction of innate IL-1, which in turn drives the induction of protective Th17 cells.

A significant new finding of our study is that Th17 cells mediate protective immunity induced by Pa through recruitment of macrophages and neutrophils to the lungs, which phagocytose and kill *B. pertussis*. Immunization with Pa was associated with rapid induction of chemokines and recruitment of neutrophils and macrophages to the lungs after *B. pertussis* challenge and these inflammatory responses and associated bacterial clearance were significantly reduced in IL-17A^−/−^ mice. We had previously reported that IL-17A and IFN-γ enhance killing of *B. pertussis* by macrophages [15]. Here, we found that IL-17A and IFN-γ also promoted killing of *B. pertussis* by neutrophils *in vitro*. Neutrophils do not appear to play a critical role in clearing a primary infection with *B. pertussis* in naïve mice, but are essential for control of *B. bronchiseptica* and play a role in controlling *B. pertussis* infection in immune mice [56], [57]. Although antibody did not significantly enhance killing of *B. pertussis* by neutrophils in our in vitro assay, our data do not rule out a role for antibodies, especially murine IgG2, in protective immunity against *B. pertussis*.

Although Pa have significantly improved safety profiles over Pw that they replaced and protect a significant percentage of children against a life threatening disease, there is an increasing incidence of pertussis in many developed countries [4]. In an attempt to limit the spread of *B. pertussis*, a number of countries have introduced booster vaccinations with Pa for 5–6 year olds, adolescents and even adults. However, a recent report has suggested protection from whooping cough in children that had received 5 doses of Pa is relatively short-lived and wanes substantially each year [6]. Furthermore, repeated boosting of Th2 or Th17 responses may not be desirable, since these responses can mediate hypersensitivity/allergy or autoimmunity when directed against allergens or self antigens respectively [58]. Indeed there is already evidence of hypersensitivity reactions in pre-school children following the fifth dose of Pa [59]. The corollary to this is that efficacy of a vaccine that relies heavily on IL-17A to confer protective immunity may be compromised in patients treated with IL-17A targeted drugs, which are in late stage clinical development for autoimmune diseases [60].

The induction of Th1 rather than Th2 responses by Pa delivered with an appropriate Th1-promoting adjuvant may not only be safer but more effective than alum-adjuvanted Pa. We found that Pw confer a high level of immunity by inducing Th1 cells, with a smaller contribution by Th17 cells. It has previously been shown that addition of the TLR9 agonist CpG to an alum-adjuvanted pertussis vaccine enhanced IgG1∶IgG2a antibodies, providing indirect evidence of enhanced Th1 responses [61]. In addition it has recently been reported that CpG enhances the protective efficacy of *B. pertussis* Ag when administered i.n. or i.p, with aluminum hydroxide [62]. However, immunization with *B. pertussis* Ag and CpG (30 µg/dose; without alum) did not enhance IFN-γ production or protective efficacy over that observed with Ag formulated with alum [62]. This is surprising given the previous reports that CpG enhances Th1 response to soluble antigens [35]. Here we found that alum-adjuvanted Pa induced weak Th1 responses, but formulation of an experimental Pa with CpG (50 µg/dose; without alum) promoted Th1 as well as Th17 responses and conferred a significantly greater level of protection against *B. pertussis*. Furthermore, protection induced with the CpG-adjuvanted experimental vaccine was significantly compromised in IFN-γ^−/−^, but retained IL-17A^−/−^ mice. These findings suggest that pertussis vaccine formulations that employ adjuvants that promote Th1 responses, such as TLR agonists, should be evaluated as a safe and more effective alternative to current alum-adjuvanted Pa for use in humans.

## Materials and Methods

### Mouse immunizations

All mice were maintained according to EU regulations and experiments were performed under licence from the Irish Department of Health and Children and with approval from the Trinity College Dublin Bioresources Ethics Committee. IL-1RI^−/−^, Nlrp3^−/−^, IL-17A^−/−^, IL-4^−/−^, IFN-γ^−/−^ and C57BL/6 WT mice were bred in house from established colonies and housed under specific pathogen free conditions. IL-17A^−/−^ mice were provided by Yoichiro Iwakura, Centre for Experimental Medicine and Systems Biology, Institute of Medical Science, University of Tokyo, Japan. The Pa used in this study was a commercially available INFANRIX (GSK; diphtheria and tetanus toxoids and acellular pertussis adsorbed to alum; the pertussis component comprising detoxified PT, FHA and pertactin). The Pw used in this study (the third international standard preparation, 88/522 from NIBSC, Herts., UK) was a thiomersal killed *B. pertussis* vaccine. Mice were immunized i.p. or i.m. (quadriceps muscle) twice (wk 0 and 4) with 0.2 human dose of Pa or Pw and were challenged with *B. pertussis* by aerosol inoculation or sacrificed 2 wks after second immunization. In experiments designed to examine the effect of CpG versus aluminium hydroxide (alum) as the adjuvant we use an experimental laboratory prepared Pa using two purified antigens, detoxified PT and FHA present in all 2 and 3-component Pa (1 and 2.5 µg/mouse respectively). The PT was GMP-grade genetically detoxified PT (PT-9K/129G), supplied by Novartis Vaccine, Siena, Italy. FHA was purchased from Kaketsuken, Kumamoto, Japan. Both preparation were highly purified, as determined by SDS gel chromatography and were free of detectable LPS. Phosphorothioate-stabilized oligodeoxynucleotide-containing CpG motifs (CpG) (5′tccatgacgttcctgatgc) was obtained from Sigma-Genosys Ltd, Cambridge, UK and used at 50 µg/mouse dose. Aluminum hydroxide (alhydrogel; referred to as alum) was obtained from Brenntag Biosector, Friederikssund, Denmark and used at 100 µg/mouse dose.

### *B. pertussis* respiratory challenge

Respiratory infection of mice was performed by aerosol challenge as previously described [63]. The course of *B. pertussis* infection was followed by performing CFU counts on lungs from groups of 4–5 mice at intervals after challenge. The lungs were aseptically removed and homogenised in 1 ml of sterile physiological saline with 1% casein on ice. Undiluted and serially diluted homogenate (100 µl) from individual lungs was spotted in triplicate onto Bordet-Gengou agar plates, and the number of CFU was calculated after 5 days incubation at 37°C. The limit of detection was approximately 0.3 log_10_ CFU per lung for groups of 4 mice at each time point (indicated by a dotted line on each CFU curve).

### T cell cytokine production

Mononuclear cells were prepared from the lungs of naive and *B. pertussis* infected mice by mechanical disruption of lung tissue [63]. Lung mononuclear cells or spleen cells (1–2×10^6^/ml) were cultured at 37°C and 5% CO_2_ with heat killed *B. pertussi*s or purified FHA. Stimulation with PMA (250 ng/ml; Sigma) and anti-mouse CD3 (1 µg/ml; Pharmingen, San Diego, USA) or medium only was used as positive and negative controls respectively. Supernatants were removed after 72 h and IL-4, IL-13, IL-17 and IFN-γ concentrations determined by two-site ELISA.

### FACS analysis

To determine cells infiltrating the lung following infection isolated lung mononuclear cells were isolated as above, washed and blocked with Fcγ block (1 µg/ml; BD Pharmingen) before surface staining with CD11b, GR1 and F4/80. Neutrophil numbers were determined by gating on GR1^+^ and CD11b^+^ while macrophages numbers were determined by gating on F4/80^+^ CD11b^+^ cells. For intracellular cytokine staining, isolated cervical lymph nodes (2×10^6^ cells/ml) were stimulated for 5 h with PMA, ionomycin in the presence of brefeldin A (5 µg/ml). Alternatively lung mononuclear cells were incubated for 1 h with brefeldin A (5 µg/ml) only. Cells were washed and blocked with Fcγ block (1 µg/ml; BD Pharmingen) before extracellular staining for surface CD3 and CD4 (BD Pharmingen). Cells were then fixed and permeabilized (Fix and Perm cell permeabilization kit; Caltag Laboratories) and stained for intracellular IL-17A and IFN-γ. Flow cytometric analysis was performed using a CyAN_ADP_ Flow Cytometer (DakoCytomation) and analysed with FlowJo software, with gating set on fluorescence minus one controls.

### *B. pertussis*-specific antibodies

Serum antibody responses to *B. pertussis* were quantified by ELISA using plate-bound heat-killed *B. pertussis* or FHA (5 µg/ml). Bound antibodies were detected using biotin-conjugated anti-mouse IgG, IgG1, IgG2a or IgG2c antibodies (Caltag) and peroxidase-conjugated streptavidin (BD Pharmingen). Antibody levels are expressed as the mean endpoint titre (± SE), determined by extrapolation of the linear part of the titration curve to 2 SE above the background value obtained with non-immune mouse serum.

### Dendritic cells

Bone marrow-derived DC were prepared by culture with GM-CSF as previously described [64]. DC were cultured with alum (125 µg/ml), Pa (0.025, 0.1 and 0.4 IU/ml) and ATP (5 mM; Sigma), or medium only, with or without the caspase-1 inhibitor Ac-YVAD-cmk (40 µM; Calbiochem). Supernatants were recovered and IL-1β concentrations determined by ELISA (R&D Systems).

### Neutrophil bactericidal assay

Neutrophils were collected from the peritoneal cavity of WT mice 18 hours following i.p. injection of 500 µl of 9% casein (Sigma) and were purified by centrifugation over 62% percoll (GE Healthcare) yielding a 97% pure population. *B. pertussis* (10^6^/test) were incubated with 10% normal mouse or immune mouse serum for 20 minutes at 37°C after which neutrophils (10^6^/test) together with 50 ng/ml of recombinant IL-17A, IL-17F (eBioscience) or IFN-γ (R&D systems) were added and incubated with shaking. After the appropriate times ice cold dH_2_O was used to lyse the cells and a CFU count determined as described above.

### Statistical analyses

One-way analysis of variance (ANOVA) was used to test for statistical significance of differences between more than two experimental groups. The student's *t* test was used for analysis when two groups were compared.

## Supporting Information

Figure S1 **Infection with** ***B. pertussis*** **induces IL-17F and IL-17A specific for FHA.** (A) Mice were exposed to an aerosol infection with *B. pertussis* and groups of 4 mice were sacrificed at 2 hr and 3, 7, 14, 21, 28 and 35 days post challenge. Lung mononuclear cells were stimulated with heat-killed *B. pertussis* (HKBp) and after 3 days of culture IL-17F was quantified in supernatants by ELISA. (B) Lung mononuclear cells (day 28 post challenge) were stimulated with inactivated pertussis toxin (PT), filamentous hemagglutinin (FHA) or HKBp and after 3 days of culture IL-17A was quantified by ELISA. Results are mean values for 4 mice per group at each time point and are representative of 3 independent experiments.
(PDF)

Figure S2 **IL-17A-producing CD4 T cells (Th17 cells) in the lungs of mice during infection with** ***B. pertussis*** **.** Naive C57BL/6 mice were exposed to an aerosol infection with *B. pertussis* and groups of 4 mice were sacrificed at the indicated time points. Lung mononuclear cells were incubated with brefeldin-A for 1 h and intracellular cytokine staining for IL-17A, together with surface staining for CD4 was performed, followed by FACS analysis. Results are expressed as absolute numbers of IL-17A^+^CD4^+^ cells in the lungs. Results are mean values for 4 mice per group at each time point and are representative of 2 independent experiments.
(PDF)

Figure S3 **IL-17A promotes CXCL1 production in the lungs during infection with** ***B. pertussis*** **.** C57BL/6 WT and IL-17A^−/−^ mice were aerosol challenged with *B. pertussis* and groups of 4 mice were sacrificed at the indicated time points. CXCL1 was quantified in lung lavage * p<0.05, ** p<0.01, *** P<0.001 IL-17A^−/−^ versus WT. Results are mean values for 4 mice per group at each time point and are representative of 2 independent experiments.
(PDF)

Figure S4 ***B. pertussis*** **-specific Th1 and Th2 cells from mice convalescing from** ***B. pertussis*** **.** Spleen cells from IFN-γ^−/−^ or WT mice that had cleared a respiratory infection with *B. pertussis* were stimulated *in vitro* with killed *B. pertussis* and IL-12 (Th1) or IL-1β and IL-23 (Th17) respectively. After 4 days of culture surviving cells were harvested and re-stimulated with PMA, ionomycin and brefeldin A and intracellular cytokine staining performed for IL-17A, IL-10 and IFN-γ. Results are representative FACS plots for 3 distinct bulk cultures preparations of *B. pertussis*-specific Th1 and Th2 cells.
(PDF)

Figure S5 **Pa generates Th17 as well as Th2 responses and protects against infection following immunization by i.p. or i.m. routes.** (A) C57BL/6 mice were immunized i.p. or i.m. (quadricep muscles) twice (0 and 4 weeks) with Pa. Two weeks after the second immunization, mice were exposed to an aerosol infection with *B. pertussis* and groups of 4 mice were sacrificed at 2 hr, 5 and 10 days post challenge. The number of CFU in the lungs were quantified at intervals after challenge. (B) Day 10 post challenge cervical lymph node cells were re-stimulated with PMA, ionomycin and brefeldin A and cells were stained for surface CD4 and intracellular IL-17 and IL-4. Results in A are mean values for 4 mice per group at each time point, results in B are sample FACS plots from 4 mice per group and are representative of 2 independent experiments.
(PDF)

Figure S6 **Pa induces IL-1β production by DC via activation of caspase-1 and Nlrp3.** Murine bone marrow-derived DC from WT or Nlrp3^−/−^ mice (B) were stimulated with a commercially available Pa (0.025, 0.1 and 0.4 IU/ml) or with alum (alum (125 µg/ml) or ATP (2.5 nM) in the presence or absence of a caspase-1 inhibitor YVAD (40 µM) (A) following 2 hr priming with LPS (100 ng/ml). After 24 hours the concentration of IL-1β in supernatants was quantified by ELISA. (C) WT mice were injected in the footpads with Pa (0.2 human dose), medium or an equivalent dose of alum (35 µg). After 4 hr, the popliteal lymph nodes were removed and homogenized and IL-1β concentrations in the homogenate determined by ELISA. Results are mean values for 4 mice per group at each time point and each panel is representative of 2 independent experiments.
(PDF)

Figure S7 **Pa induces IL-1β and IL-17 production and protective immunity against** ***B. pertussis in vivo*** **largely independent of Nlrp3.** WT and Nlrp3^−/−^ mice were immunized twice (0 and 28 days) with Pa or PBS. 14 days after the second immunization, mice were challenged by exposure to an aerosol of live *B. pertussis*. (A) The number of CFU in the lungs were quantified at the indicated intervals after challenge. **p<0.01 versus WT. (B) IL-17 production detected by ELISA in cervical lymph nodes removed 5 days after challenge and re-stimulated *in vitro* with heat killed *B. pertussis* (HKBp) or medium only. (C) Cervical lymph node cells were re-stimulated with PMA, ionomycin and brefeldin A and cells were stained for surface CD4 and intracellular IL-17. D) WT and Nlrp3^−/−^ mice were challenged by exposure to an aerosol of live *B. pertussis*. After 3 days IL-1β was quantified in lung homogenates by ELISA. Results in A, B and D are mean values for 4 mice per group at each time point and each panel is representative of 2 independent experiments. Results in C are representative FACS plots for 4 mice per group from 2 experiments.
(PDF)

Figure S8 **Enhanced macrophages recruitment to the lungs post** ***B. pertussis*** **challenge of mice immunized with Pa and CPG is mediated by Th1 cells.** WT, IL-17A^−/−^ or IFN-γ^−/−^ mice were immunized i.p. twice with a laboratory-prepared Pa formulated with CpG or WT mice were immunized with PBS. Mice were challenged by exposure to an aerosol of live *B. pertussis* 14 days after the second immunization. Three days after challenge, the number of macrophages in the lungs were quantified by FACS staining for F4/80^+^CD11b^+^ cells ; *p<0.05, versus WT+PBS. Results are mean values for 4 mice per group and are representative of 2 independent experiments.
(PDF)
