# Supplementary figures and images for: Relative Contribution of Th1 and Th17 Cells in Adaptive Immunity to Bordetella pertussis: Towards the Rational Design of an Improved Acellular Pertussis Vaccine
Source: PLoS Pathog. 2013 Apr 4;9(4):e1003264. doi: 10.1371/journal.ppat.1003264 (PMC3617212; doi:10.1371/journal.ppat.1003264)

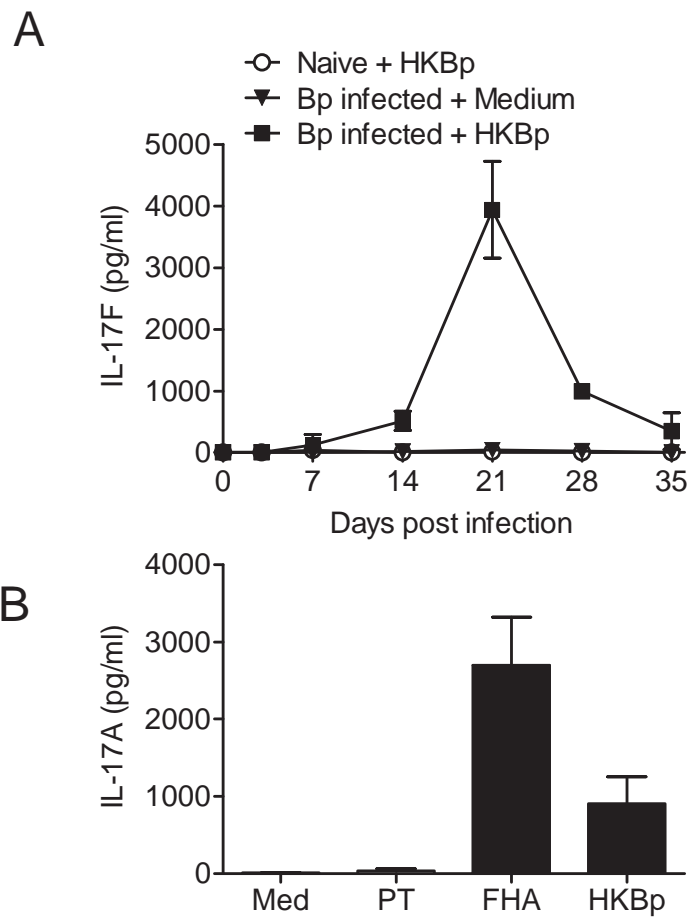

Figure S1

Supplement: Figure S1 — Infection with B. pertussis induces IL-17F and IL-17A specific for FHA. (A) Mice were exposed to an aerosol infection with B. pertussis and groups of 4 mice were sacrificed at 2 hr and 3, 7, 14, 21, 28 and 35 days post challenge. Lung mononuclear cells were stimulated with heat-killed B. pertussis (HKBp) and after 3 days of culture IL-17F was quantified in supernatants by ELISA. (B) Lung mononuclear cells (day 28 post challenge) were stimulated with inactivated pertussis toxin (PT), filamentous hemagglutinin (FHA) or HKBp and after 3 days of culture IL-17A was quantified by ELISA. Results are mean values for 4 mice per group at each time point and are representative of 3 independent experiments. (PDF) [file ppat.1003264.s001.pdf]

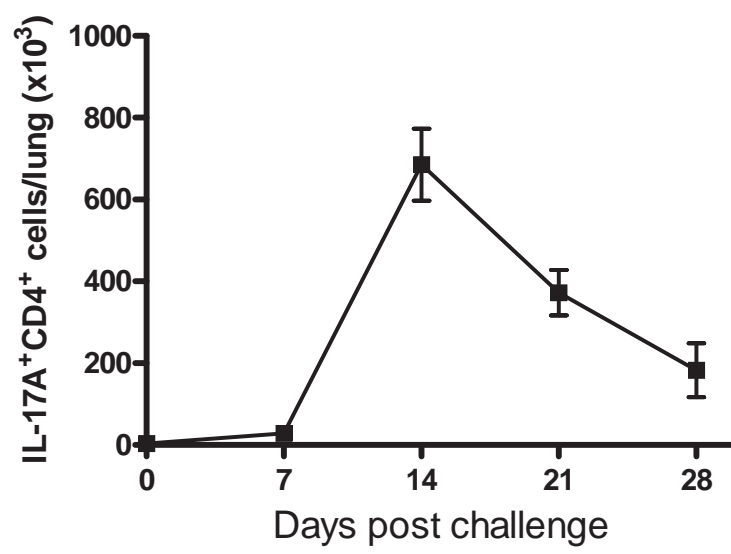

Figure S2

Supplement: Figure S2 — IL-17A-producing CD4 T cells (Th17 cells) in the lungs of mice during infection with B. pertussis . Naive C57BL/6 mice were exposed to an aerosol infection with B. pertussis and groups of 4 mice were sacrificed at the indicated time points. Lung mononuclear cells were incubated with brefeldin-A for 1 h and intracellular cytokine staining for IL-17A, together with surface staining for CD4 was performed, followed by FACS analysis. Results are expressed as absolute numbers of IL-17A+CD4+ cells in the lungs. Results are mean values for 4 mice per group at each time point and are representative of 2 independent experiments. (PDF) [file ppat.1003264.s002.pdf]

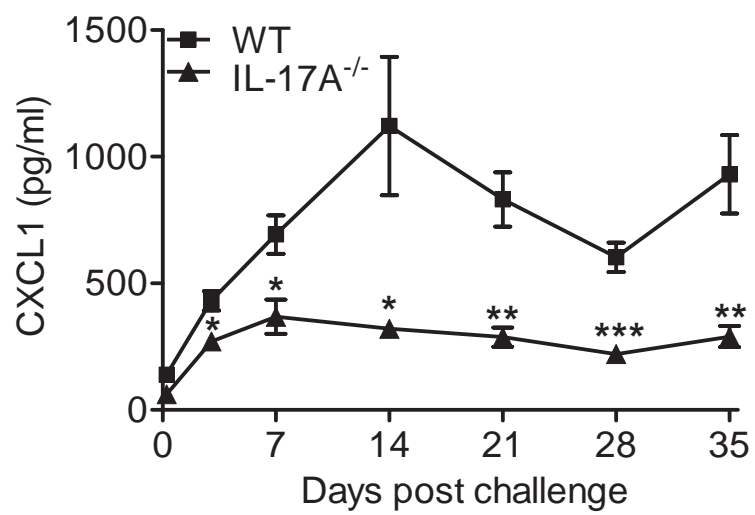

Figure S3

Supplement: Figure S3 — IL-17A promotes CXCL1 production in the lungs during infection with B. pertussis . C57BL/6 WT and IL-17A−/− mice were aerosol challenged with B. pertussis and groups of 4 mice were sacrificed at the indicated time points. CXCL1 was quantified in lung lavage * p<0.05, ** p<0.01, *** P<0.001 IL-17A−/− versus WT. Results are mean values for 4 mice per group at each time point and are representative of 2 independent experiments. (PDF) [file ppat.1003264.s003.pdf]

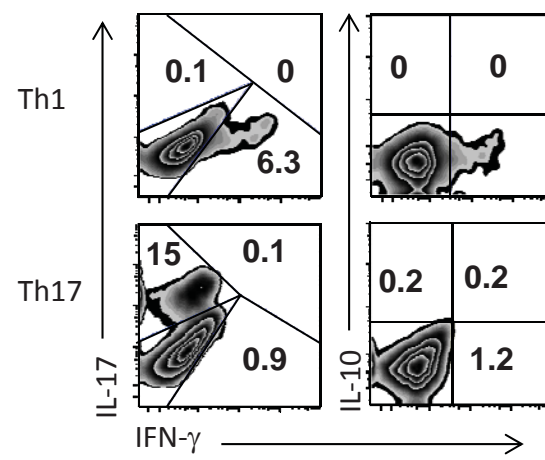

Figure S4

Supplement: Figure S4 — B. pertussis -specific Th1 and Th2 cells from mice convalescing from B. pertussis . Spleen cells from IFN-γ−/− or WT mice that had cleared a respiratory infection with B. pertussis were stimulated in vitro with killed B. pertussis and IL-12 (Th1) or IL-1β and IL-23 (Th17) respectively. After 4 days of culture surviving cells were harvested and re-stimulated with PMA, ionomycin and brefeldin A and intracellular cytokine staining performed for IL-17A, IL-10 and IFN-γ. Results are representative FACS plots for 3 distinct bulk cultures preparations of B. pertussis-specific Th1 and Th2 cells. (PDF) [file ppat.1003264.s004.pdf]

**A**

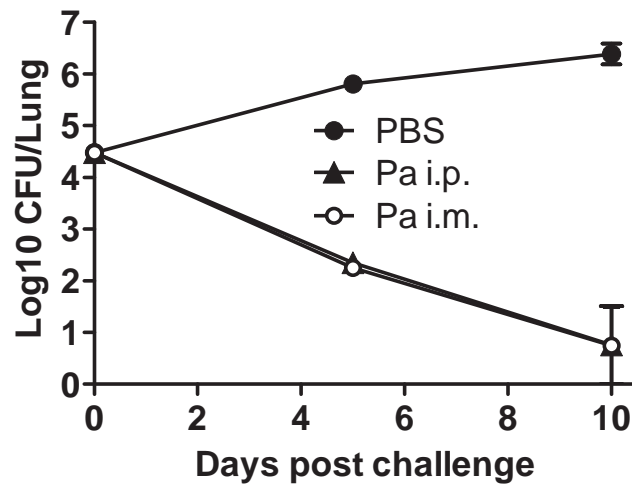

**B**

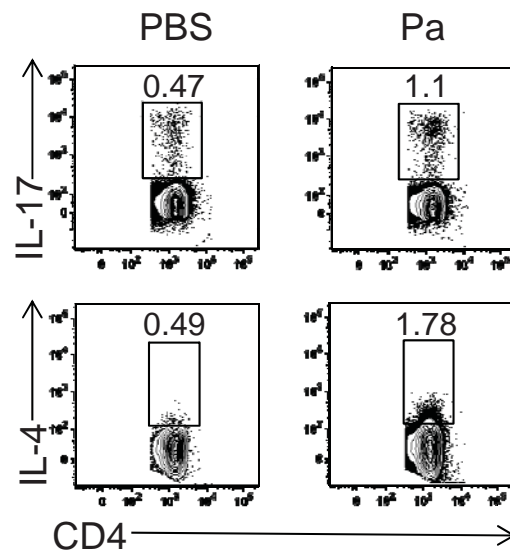

Figure S5

Supplement: Figure S5 — Pa generates Th17 as well as Th2 responses and protects against infection following immunization by i.p. or i.m. routes. (A) C57BL/6 mice were immunized i.p. or i.m. (quadricep muscles) twice (0 and 4 weeks) with Pa. Two weeks after the second immunization, mice were exposed to an aerosol infection with B. pertussis and groups of 4 mice were sacrificed at 2 hr, 5 and 10 days post challenge. The number of CFU in the lungs were quantified at intervals after challenge. (B) Day 10 post challenge cervical lymph node cells were re-stimulated with PMA, ionomycin and brefeldin A and cells were stained for surface CD4 and intracellular IL-17 and IL-4. Results in A are mean values for 4 mice per group at each time point, results in B are sample FACS plots from 4 mice per group and are representative of 2 independent experiments. (PDF) [file ppat.1003264.s005.pdf]

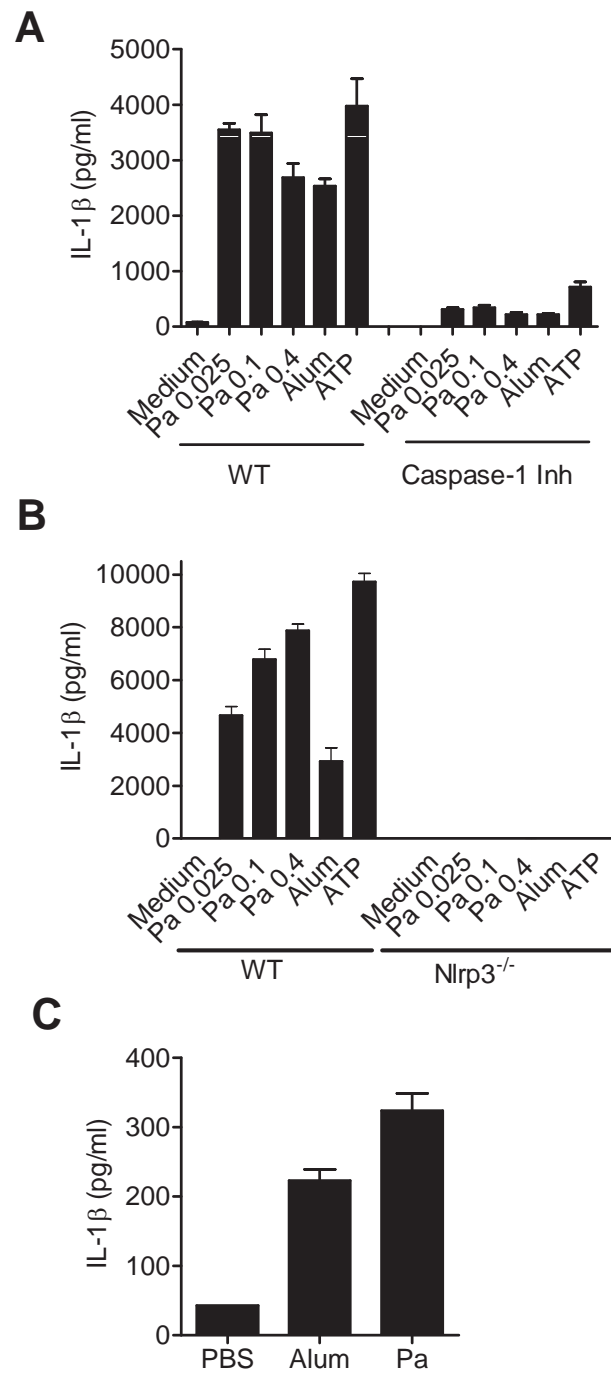

Figure S6

Supplement: Figure S6 — Pa induces IL-1β production by DC via activation of caspase-1 and Nlrp3. Murine bone marrow-derived DC from WT or Nlrp3−/− mice (B) were stimulated with a commercially available Pa (0.025, 0.1 and 0.4 IU/ml) or with alum (alum (125 µg/ml) or ATP (2.5 nM) in the presence or absence of a caspase-1 inhibitor YVAD (40 µM) (A) following 2 hr priming with LPS (100 ng/ml). After 24 hours the concentration of IL-1β in supernatants was quantified by ELISA. (C) WT mice were injected in the footpads with Pa (0.2 human dose), medium or an equivalent dose of alum (35 µg). After 4 hr, the popliteal lymph nodes were removed and homogenized and IL-1β concentrations in the homogenate determined by ELISA. Results are mean values for 4 mice per group at each time point and each panel is representative of 2 independent experiments. (PDF) [file ppat.1003264.s006.pdf]

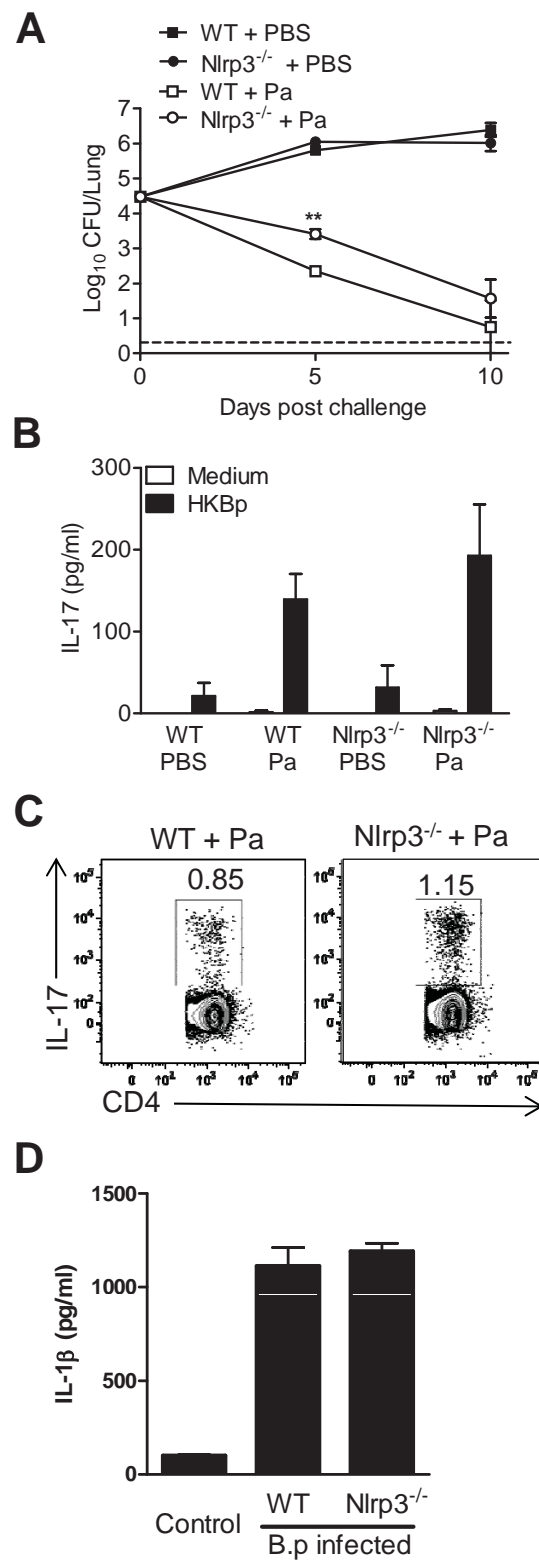

Figure S7

Supplement: Figure S7 — Pa induces IL-1β and IL-17 production and protective immunity against B. pertussis in vivo largely independent of Nlrp3. WT and Nlrp3−/− mice were immunized twice (0 and 28 days) with Pa or PBS. 14 days after the second immunization, mice were challenged by exposure to an aerosol of live B. pertussis. (A) The number of CFU in the lungs were quantified at the indicated intervals after challenge. **p<0.01 versus WT. (B) IL-17 production detected by ELISA in cervical lymph nodes removed 5 days after challenge and re-stimulated in vitro with heat killed B. pertussis (HKBp) or medium only. (C) Cervical lymph node cells were re-stimulated with PMA, ionomycin and brefeldin A and cells were stained for surface CD4 and intracellular IL-17. D) WT and Nlrp3−/− mice were challenged by exposure to an aerosol of live B. pertussis. After 3 days IL-1β was quantified in lung homogenates by ELISA. Results in A, B and D are mean values for 4 mice per group at each time point and each panel is representative of 2 independent experiments. Results in C are representative FACS plots for 4 mice per group from 2 experiments. (PDF) [file ppat.1003264.s007.pdf]

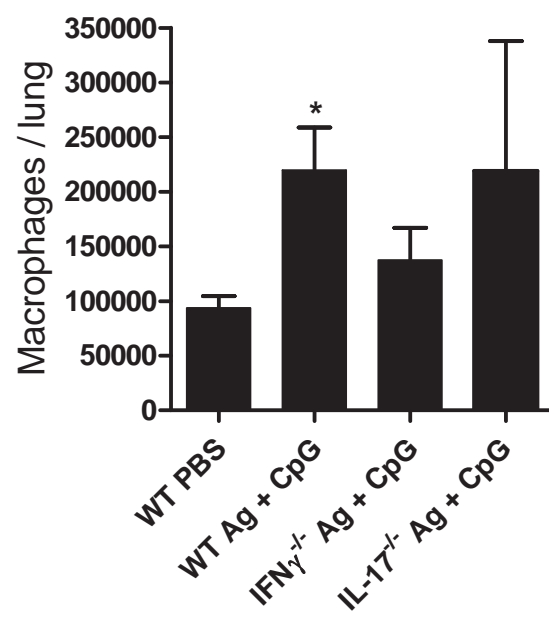

Figure S8

Supplement: Figure S8 — Enhanced macrophages recruitment to the lungs post B. pertussis challenge of mice immunized with Pa and CPG is mediated by Th1 cells. WT, IL-17A−/− or IFN-γ−/− mice were immunized i.p. twice with a laboratory-prepared Pa formulated with CpG or WT mice were immunized with PBS. Mice were challenged by exposure to an aerosol of live B. pertussis 14 days after the second immunization. Three days after challenge, the number of macrophages in the lungs were quantified by FACS staining for F4/80+CD11b+ cells ; *p<0.05, versus WT+PBS. Results are mean values for 4 mice per group and are representative of 2 independent experiments. (PDF) [file ppat.1003264.s008.pdf]
